# Association between Coagulation Function and Cerebral Microbleeds in Ischemic Stroke Patients with Atrial Fibrillation and/or Rheumatic Heart Disease

**DOI:** 10.14336/AD.2016.0715

**Published:** 2017-04-01

**Authors:** Junfeng Liu, Deren Wang, Yao Xiong, Bian Liu, Jing Lin, Shihong Zhang, Bo Wu, Chenchen Wei, Ming Liu

**Affiliations:** Stroke Clinical Research Unit, Department of Neurology, West China Hospital, Sichuan University, Chengdu, 610041 China

**Keywords:** Coagulation function, Fibrinogen, Cerebral microbleeds, Ischemic stroke

## Abstract

Cerebral microbleeds (CMBs), which indicate hemorrhage-prone disease, may associate with hemostatic abnormalities, but the association between CMBs and coagulation function is uncertain. We aimed to examine this possible association. The following coagulation function indicators were evaluated in 85 consecutive ischemic stroke patients diagnosed with atrial fibrillation and/or rheumatic heart disease: prothrombintime (PT), activated partial thromboplastin time (APTT), and levels of D-dimer and fibrinogen. Indicators were assessed within 24 h after admission. CMBs were identified based on published criteria by two experienced stroke neurologists working independently. PT, APPT, and levels of D-dimer and fibrinogen were compared between patients with and without CMBs using univariate and multivariate analysis. CMBs were detected in 48 patients (56.5%), and fibrinogen levels in these patients were independently and significantly higher than in patients without CMBs after adjustment (OR 2.16, 95% CI 1.20-3.90, *P*=0.01), whereas the two types of patients did not differ significantly in PT, APPT, or D-dimer levels. The presence of CMBs in ischemic stroke patients with atrial fibrillation and/or rheumatic heart disease is associated with elevated levels of fibrinogen. Larger prospective studies are needed to verify this association and explore the mechanisms involved.

Cerebral microbleeds (CMBs) are one kind of cerebral small vessel disease [1]. They manifest as small, round lesions showing signal loss on T2*-weighted gradient echo (GRE) or susceptibility-weighted magnetic resonance imaging, which is due to perivascular hemosiderin [2]. Strictly lobar CMBs are associated with cerebral amyloid angiopathy, and deep CMBs are associated with hypertensive arteriopathy [3-5].

CMBs occur in many patients with ischemic stroke, and primary intracerebral hemorrhage (ICH); CMBs are also associated with elevated risk of spontaneous ICH, and symptomatic ICH after intravenous thrombolysis [6-8]. CMBs are thought to indicate hemorrhage-prone disease in the brain [9], but whether such bleeding is associated with hemostatic abnormalities or vascular lesions needs to be clarified.

Several studies have suggested a link between CMBs and coagulation dysfunction. Prodan et al [10] found that the presence of CMBs in patients with non-lacunar ischemic stroke is associated with lower levels of coated-platelets, which are involved in thrombin generation and thereby help drive coagulation [11]. CMBs were identified in a 62-year-old Spanish male with disseminated intravascular coagulation, a serious disease related to coagulation dysfunction [12]. These sporadic reports suggest an association between CMBs and compromised coagulation function, but more systematic studies are needed.

Therefore, the present research was undertaken to compare ischemic stroke patients with and without CMBs in terms of several indicators of coagulation function, including prothrombin time, activated partial thrombo-plastin time, and levels of D-dimer and fibrinogen.

## MATERIAS AND METHODS

This research was conducted using data from the National Natural Science Foundation of China project "Study on small vessel pathological mechanism of cerebral hemorrhage after cardioembolic stroke using susceptibility-weighted MR imaging markers’’. The research was approved by the Medical Ethics Committee of West China Hospital, Sichuan University. Written informed consent was obtained from participants or their next of kin.

The present study prospectively enrolled consecutive ischemic stroke patients with atrial fibrillation and/or rheumatic heart disease who were admitted to West China Hospital of Sichuan University (Chengdu, China) between January 2014 and March 2016, within one month after stroke onset. To participate in the study, patients had to undergo complete susceptibility-weighted magnetic resonance imaging within 7 days after admission. The diagnosis of ischemic stroke, made based on World Health Organization criteria [13], had to be confirmed based on computed tomography scanning or magnetic resonance imaging [14]. Atrial fibrillation was defined as a history of persistent or paroxysmal atrial fibrillation, documented by electrocardiography during admission (24-hour or not) [15]. Rheumatic heart disease was diagnosed according to criteria in the International Classification of Diseases (10th edition) and confirmed by echocardiography [16]. Patients were excluded from the study if their coagulation function was not analyzed within 24 h after admission by measuring prothrombintime (PT), activated partial thromboplastin time (APTT), and levels of D-dimer and fibrinogen.

A standardized form was used to collect the following patient information: demographic characteristics (age and gender); risk factors (hypertension, diabetes mellitus, hyperlipidemia, history of transient ischemic attacks and stroke, current smoking and alcohol consumption); stroke severity on admission (score on the National Institutes of Health Stroke Scale [NIHSS]) [17]; and renal impairment, which was defined as an estimated glomerular filtration rate < 60ml/min/1.73m^2^ [18-19], based on medical history or prospective measurement.

Coagulation function was assessed using routine laboratory analysis of blood samples taken within 24 h after admission. Levels of D-dimer and fibrinogen were measured using, respectively, acalibrated SYSMEX7000 analyzer (Sysmex Corporation, Hyogo, Japan) and STA-R analyzer (Evolution, Stago, France). The reference range for D-dimer was <0.55 mg/L fibrinogen equivalent units (FEU), and the reference range for fibrinogen was 2-4g/L. PT, APTT, and levels of serum cholesterol, triglycerides (TG), high-density lipoprotein (HDL), and low-density lipoprotein (LDL) were also measured. All these analyses were performed in the Department of Laboratory Medicine of West China Hospital, Sichuan University, which has been fully accredited by the Chinese Ministry of Health and the College of American Pathologists’ Laboratory Accreditation Program [20].

All participants were imaged by fluid-attenuated inversion recovery and susceptibility-weighted magnetic resonance imaging at the Huaxi MR Research Center within 7 days after admission using a dedicated 3-T imaging system (Siemens Trio). The following operating parameters were used to perform susceptibility-weighted magnetic resonance imaging, and they remained unchanged throughout the study: type of echo sequence, multislice gradient; repetition time, 207ms; echo time, 20 ms; flip angle, 15°; slice thickness, 2 mm; number of slices, 60; interslice gap, 0 mm; coverage, entire brain; field of view, 220 x 173 mm; matrix, 256 x 256. The following operating parameters were used to perform fluid-attenuated inversion recovery magnetic resonance imaging, and they remained unchanged throughout the study: repetition time, 6000 ms; echo time, 100 ms; flip angle, 90°; slice thickness, 5 mm; number of slices, 21; interslice gap, 1.5 mm; field of view, 220 x 200 mm; matrix, 256 x 256.

CMBs were defined as small, circular, homogeneous lesions appearing throughout the brain with very low signal intensity and diameter <10 mm on susceptibility-weighted magnetic resonance images [5,21]. CMBs were classified based on location as (a) strictly lobar CMBs (b) deep or infratentorial CMBs with or without concomitant lobar CMBs [2,3]. Two neurologists blinded to clinical data independently assessed each patient for the presence of CMBs. A third neurologist arbitrated in case of disagreement, and a consensus decision was reached. Inter-rater reliability for determining the presence and location of CMBs was 0.73.

Data for continuous variables are reported as mean ± standard deviation or as median (interquartile range [IQR]), while data for categorical variables are reported as frequencies and percentages. Statistical significance of inter-group differences was assessed using the χ^2^ test, Fisher’s exact test (categorical data), or the *t* test or Mann-Whitney U test (continuous data), as appropriate. Possible correlations of CMB occurrence with patient variables and/or coagulation function were explored using multivariate logistic regression. When appropriate, results were reported using odds ratios (ORs) and 95% confidence intervals (95%CIs). All data were analyzed using The Statistical Package for the Social Sciences 20.0 (SPSS; IBM, Chicago, IL, USA). The threshold of significance was defined as *p* < 0.05.

## RESULTS

The study enrolled a consecutive sample of 109 ischemic stroke patients, along with atrial fibrillation and/or rheumatic heart disease, who were admitted to our hospital within one month of stroke onset and underwent susceptibility-weighted magnetic resonance imaging within 7 days after admission. Of these enrolled patients, 24 were excluded because their coagulation function was not analyzed within 24 h after admission. The remaining 85 patients (35.3% male) were included in the final analysis. Their mean age was 68.96 ± 11.90 yr (ranges from 42 to 92). Most patients (67) had only atrial fibrillation, 4 of them had only rheumatic heart disease, and 14 of them had both conditions. Just over half (48, 56.5%) showed evidence of at least one CMB (ranges from 1-10): of these patients, 26 (54.2%) had strictly lobar CMBs, while the remaining 22 (45.8%) had deep or infratentorial CMBs with or without concomitant lobar CMBs.

**Table 1:** Baseline characteristics of ischemic stroke patients with atrial fibrillation and/or rheumatic heart disease, stratified by presence or absence of cerebral microbleeds

Characteristic	Total N = 85	CMBs N = 48	No CMBs N = 37	*p*
**Male**	30 (35.3)	19 (39.6)	11 (29.7)	0.35
**Age, yr**	68.94 ± 11.90	70.98 ± 10.50	66.30 ± 13.18	0.07
**Current smoking**	69 (81.2)	39 (81.3)	30 (81.1)	0.99
**Current alcohol consumption**	73 (85.9)	42 (87.5)	31 (83.8)	0.83
**Hypertension**	38 (44.7)	25 (52.1)	13 (35.1)	0.12
**Diabetesmellitus**	25 (29.4)	12 (25.0)	13 (35.1)	0.31
**Renal impairment**	15 (17.6)	9 (18.8)	6 (16.2)	0.76
**Previous TIA/stroke**	17 (20.0)	9 (18.8)	8 (21.6)	0.74
**NIHSS score on admission**	9 (4-13)	6.5 (3-10)	12 (6-16)	**0.03**
**Total cholesterol, mmol/L**	4.03 ± 0.91	4.12 ± 0.97	3.91 ± 0.83	0.29
**TG, mmol/L**	1.49 ± 1.35	1.60 ± 1.64	1.35 ± 0.83	0.41
**HDL, mmol/L**	1.39 ± 0.39	1.41 ± 0.40	1.36 ± 0.38	0.61
**LDL, mmol/L**	2.26±0.80	2.36 ± 0.87	2.13 ± 0.69	0.18
**PT, s**	12.54 ± 1.61	12.59 ± 1.75	12.47 ± 1.44	0.73
**APTT, s**	28.43 ± 7.02	29.13 ± 8.93	27.54 ± 3.01	0.31
**D-dimer, mg/L**	2.51 ± 5.14	2.73 ± 5.70	2.22 ± 4.36	0.65
**Fibrinogen, g/L**	3.02 ± 1.03	3.24 ± 1.14	2.73 ± 0.78	**0.02**

Values are n (%), median (IQR) or mean ± SD.

*Abbreviations:* APTT, activated partial thromboplastin time; CMBs, cerebral microbleeds; HDL, high-density lipoprotein; hs-cTnT, high-sensitivity cardiac troponin T; LDL, low-density lipoprotein; NIHSS, National Institutes of Health Stroke Scale; PT, prothrombintime; TG,

The characteristics of study participants stratified by presence or absence of CMBs are shown in Table 1, which reports coagulation function indicators as continuous variables. Patients with CMBs were more likely to have a lower NIHSS score on admission than patients without CMBs (*p* = 0.03), as well as a significantly higher level of fibrinogen (*p* =0.02). Patients with CMBs tended to have longer PT (*p* = 0.73), longer APTT (*p* = 0.31) and a higher level of D-dimer (*p* = 0.65).

Multivariate analysis was performed using a model that adjusted for age, gender and baseline variables differing significantly between patients with or without CMBs, *i.e*. NIHSS on admission. The model identified fibrinogen level as an independent risk factor for CMB occurrence (OR 2.16, 95% CI 1.20-3.90, *p* = 0.01; Table 2).

**Table 2:** Multivariate analysis to identify coagulation function indicators independently associated with CMB occurrence^*^

	OR	95% CI	*p*
**PT, s**	1.06	0.79-1.44	0.69
**APTT, s**	1.04	0.95-1.14	0.37
**D-dimer, mg/L**	1.02	0.92-1.13	0.74
**Fibrinogen, g/L**	2.16	1.20-3.90	0.01

* Data wereadjusted for age, gender, and NIHSS score on admission.

*Abbreviations:* APTT, activated partial thromboplastin time; CI, confidence interval; CMB, cerebral microbleeds; NIHSS, National Institutes of Health Stroke Scale; OR, odds ratio; PT, prothrombintime

## DISCUSSION

This prospective study assessed possible relationships between indicators of coagulation function and the presence of CMBs in ischemic stroke patients with atrial fibrillation and/or rheumatic heart disease. Our results suggest that fibrinogen levels are independently associated with risk of CMBs, while PT, APTT and D-dimer levels are not. To our knowledge, this is the first study to examine possible correlations between coagulation function and CMBs.

Why fibrinogen levels may be associated with risk of CMBs is unclear. Fibrinogen, a critical element of platelet cross-linking and clot formation, plays an important role in arterial thrombosis [22]. A study in Japan [23] has linked serum fibrinogen levels with intima-media thickness and atherosclerosis. Furthermore, fibrinogen is generated and released when systemic inflammation occurs [24], and both inflammation and atherosclerosis have been linked to CMB formation [25]. Taken together, these data suggest that elevated fibrinogen levels may increase risk of CMBs through their association with inflammation and atherosclerosis.

The present study involved a relatively small, highly specific patient population, so future prospective studies with larger cohorts are needed to verify and extend our finding of an association between elevated fibrinogen levels and risk of CMB occurrence. Those studies should examine whether and how elevated fibrinogen levels may contribute to CMB formation as well as to other small cerebral vascular diseases.

### Conclusion

Elevated serum fibrinogen concentration in ischemic stroke patients with atrial fibrillation and/or rheumatic heart disease may be associated with CMB formation. Future studies should verify this association and explore how elevated fibrinogen may contribute to CMB formation.
